# Spatial differentiation and coupling between village development intensity and landscape pattern of 100 villages in Anhui, China

**DOI:** 10.1038/s41598-025-88849-w

**Published:** 2025-02-11

**Authors:** Bohang Zhang, Jiahan Zhou, Lihua Chen

**Affiliations:** 1 School of Natural Sciences, The University of Manchester, Manchester, M13 9PL UK; 2 Department of Urban Planning, Hefei University of Technology, Hefei, 230601 China

**Keywords:** Village spatial development intensity, Landscape pattern, Spatial differentiation, Coupling relationship, Anhui Province, China, Ecology, Environmental sciences, Environmental social sciences

## Abstract

Spatial development and landscape pattern are fundamental elements of the land system of village. Analysing the spatial differentiation and coupling relationship between spatial development intensity and landscape pattern is of great significance for the development and protection of village land resources. In order to address the current research lack on the coupling response between village spatial development intensity and landscape pattern, a technical method for analysing the spatial differentiation and coupling relationship between village spatial development intensity and landscape pattern is constructed based on the methods of village spatial development intensity model, landscape pattern index, bivariate spatial autocorrelation model, coupling degree and coupling coordination degree model. Taking 100 villages in Anhui Province, China as an example, the spatial distribution characteristics and coupling characteristics of village spatial development intensity and landscape pattern are analysed. The results show that there are obvious regional differences in the spatial distribution of village spatial development intensity and landscape pattern in Anhui Province. The village spatial development intensity shows a pattern of the Northern Anhui plain region (NAPR) > along the Yangtze River plain region (YRPR) > Jiang-huai Hilly region (JHHR) > Southern Anhui mountainous region (SAMR) > Western Anhui mountainous region (WAMR). The village landscape pattern in NAPR and YRPR are high fragmentation, while the village in JHHR has the lowest fragmentation, and the villages in SAMR and WAMR show relatively low fragmentation. The spatial coupling relationship between village spatial development intensity and landscape pattern is mainly characterised by high-high clustering and low-high clustering. The coupling coordinated development of villages in NAPR is the best, followed by YRPR, JHHR and SAMR, and WAMR is the worst. There is only a significant multi-linear relationship between village landscape pattern and multiple spatial development intensity indicators in WAMR and NAPR. The spatial differentiation and coupling relationship are influenced by both natural geographical factors and human activity factors. Finally, the study puts forward some targeted countermeasures and suggestions. The research results can provide theoretical method and practical application reference for village land space development and protection and village planning.

## Introduction

Spatial development intensity reflects the degree of land use and the intensity of human activities in a given area, serving as a comprehensive measure of regional land development and spatial growth^1–3^. It is a direct indicator of land development and spatial development, characterized by multi-level, multi-purpose, multi-element, as well as complex and dynamic attributes^4^. Village spatial development intensity, specifically, refers to the degree of land development and utilization within a village, indicating the extent to which the village’s natural geographical environment is influenced by human activities. As a direct manifestation of human activity intensity, spatial development intensity has become a focal area of global change research^5–7^, typically expressed through metrics such as the magnitude and density of land development^8^. Landscape patterns represent the cumulative impact of human activities on the natural geographical environment^9^. These patterns primarily include the spatial distribution of different land-use types, as well as the size and shape of landscape patches^10–12^. Spatial development drives land-use changes, which in turn lead to corresponding changes in landscape patterns. Different levels of spatial development intensity result in distinct landscape pattern characteristics. Therefore, exploring the intrinsic coupling relationship between spatial development intensity and landscape patterns is of great significance.

## Research background and research objectives

### Research background

Since the 21st century, China has experienced rapid urbanization and industrialization, which has driven an accelerated phase of rural spatial development. Over the past two decades, this swift development in village spaces has led to two primary issues. First, spatial development activities have intensified in certain villages, increasing development intensity significantly. While this has promoted rapid economic and social growth, it has also altered the traditional landscape structure of these villages. For instance, villages located in plains with well-established infrastructure, such as roads, have seen their landscapes progressively urbanize through years of spatial development, resulting in a gradual disappearance of traditional village appearances. Conversely, villages situated in mountainous areas with poor infrastructure conditions face challenges in spatial development. Although their traditional landscape patterns remain preserved due to lower development intensity, this has, in turn, hindered their economic and social progress. In October 2017, the Chinese government introduced the National Rural Revitalization Strategy with the primary aim of addressing these regional disparities in village spatial development and achieving a balanced development that harmonizes spatial growth with landscape preservation. Under the comprehensive implementation of the Rural Revitalization Strategy, it has become increasingly crucial to understand the distribution characteristics of spatial development intensity and landscape structure in rural areas. Identifying and analyzing the coupling relationship between these two aspects holds significant theoretical, methodological, and practical value for advancing the rural revitalization strategy, coordinating village land use planning and conservation, and developing effective village planning initiatives.

### Literature review

The research of spatial development intensity mainly focusses on the evaluation and optimisation of land development intensity^13–17^, the study of the special land development intensity^18–22^, and the relationship between land development intensity and habitat quality^23–26^. Research on landscape pattern focusses on the spatial characteristics, temporal characteristics and driving factors of landscape patterns^27–33^. In terms of research methods, GIS spatial analysis technology, comprehensive index method and landscape pattern index are the most commonly used methods^34^. The research scope mainly focusses on national, regional, provincial and urban scales^35–39^. Overall, there are still gaps in existing research. First, in terms of research subjects, most studies focus on meso- and macro-scales, such as cities and regions. Villages, as micro-level units within the territorial spatial system, have received little attention in studies examining the relationship between spatial development intensity and landscape patterns. Second, regarding research content, existing studies primarily adopt a single perspective, focusing either on spatial development intensity or on landscape pattern characteristics. They are often centered on topics such as urban and regional land-use types or the evolution of landscape patterns. However, at the village scale, the spatial differentiation of development intensity and landscape patterns, as well as their coupling relationships, remain underexplored. Finally, empirical studies focusing on villages remain relatively scarce, particularly those comparing spatial development and landscape patterns across villages located in different geographical divisions.

### Research objectives

Given these research gaps, this study aims to explore the spatial differentiation and coupling relationships between village spatial development intensity and landscape patterns at the micro scale. The first objective is to establish a technical methodology framework for analyzing the spatial differentiation and coupling relationships. The second objective is to examine the spatial distribution characteristics of village spatial development intensity and landscape patterns. The third objective is to investigate the coupling relationships between them, including both spatial and non-spatial coupling relationships.

This study contributes to the research field in several ways. First, by focusing on villages, it addresses the lack of micro-scale studies in the current research field. Second, it establishes an integrated framework to evaluate the spatial differentiation and coupling relationships between village spatial development intensity and landscape patterns. In particular, the study of coupling relationships provides essential decision-support information for identifying village spatial development models and scientifically formulating village spatial plans, which are critical for achieving high-quality and sustainable village development. Consequently, this research not only enriches the theoretical framework in the field but also offers significant practical value. Third, this study conducts a detailed empirical analysis of 100 villages from different geomorphic divisions in Anhui Province, China. By integrating GIS spatial analysis techniques, landscape pattern indices, spatial autocorrelation models, and coupling degree and coupling coordination degree models, it examines the spatial differentiation and coupling relationships between village spatial development intensity and landscape patterns. Furthermore, the study explores the influencing factors and mechanisms of these coupling relationships and provides a detailed comparative analysis of research findings across villages in different geomorphic divisions. The findings provide theoretical and methodological references for studies on spatial development and landscape patterns in villages, both in China and globally, while also offering practical guidance for village land-use development, conservation, and spatial planning.

## Theoretical basis

### Basic concepts

Spatial development intensity and landscape pattern are two fundamental attributes of regional spatial systems, closely interconnected in a complementary relationship. Spatial development intensity serves as the “surface” of the regional spatial system, outwardly reflecting the degree of spatial development and utilization within a geographic area. This includes changes in land-use types, road network density, and other visible spatial transformations, serving as the spatial foundation upon which landscape patterns evolve. Conversely, the landscape pattern represents the “core” of the spatial system, encompassing the forms, combinations, and arrangements of diverse landscape types resulting from shifts in development intensity. It emphasizes spatial heterogeneity, ecological processes, and scale interrelations, illustrating the spatial dynamics and responses of landscape units. In this way, it provides a highly refined and abstract reflection of regional spatial structure and functional characteristics.

### Coupling relationships

**Fig. 1:**
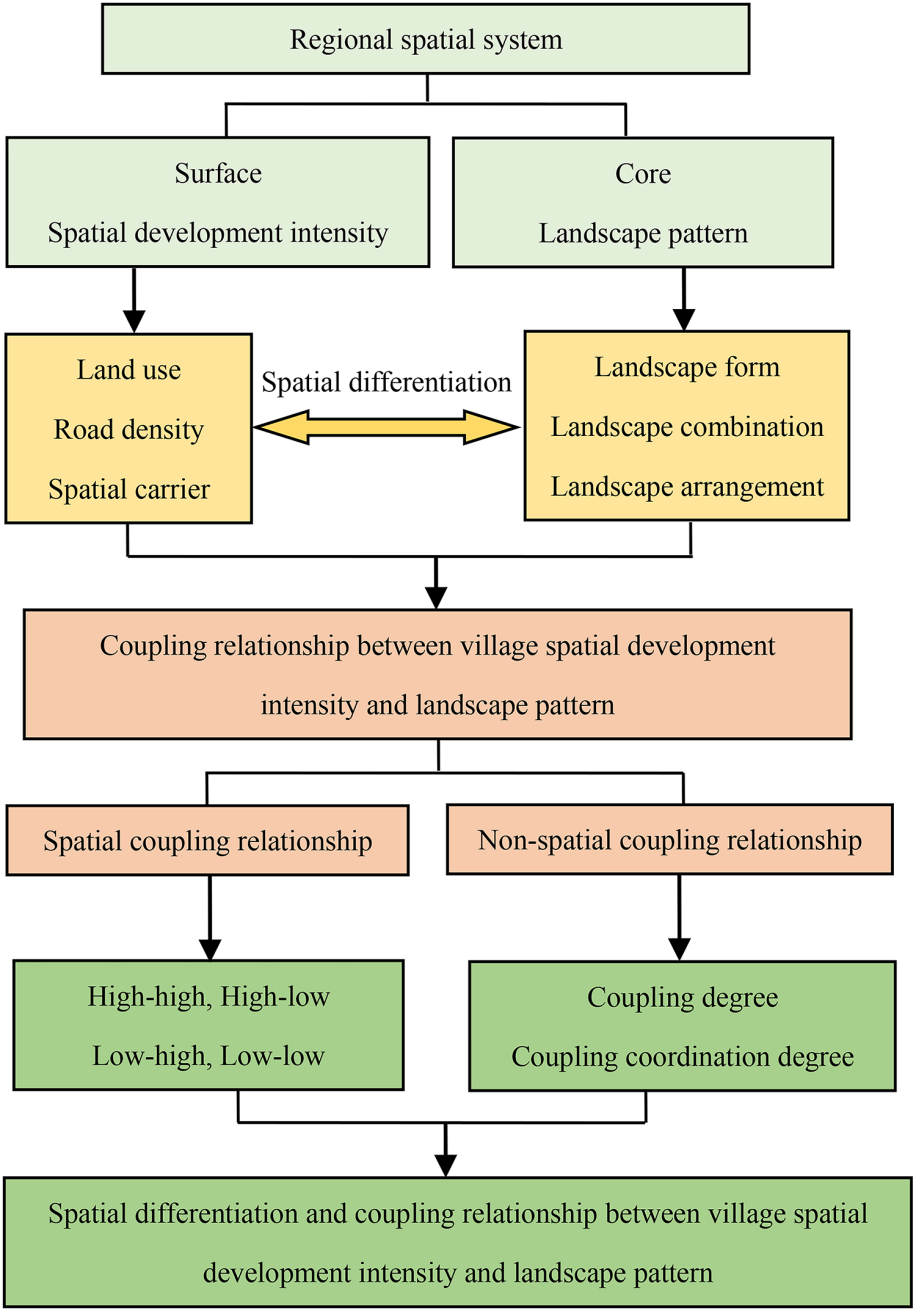
The schematic diagram of the theoretical framework.

Changes in spatial development intensity lead to corresponding shifts in landscape patterns, altering the original landscape configuration, types, and hierarchy. Ideal spatial development can preserve the uniqueness of a region’s original landscape structure while optimizing the overall landscape pattern. For instance, it may reduce landscape fragmentation and patch isolation, increase average patch size, and thereby achieve a dynamic balance and integration between development intensity and landscape pattern. The coupling relationship between spatial development intensity and landscape pattern includes both spatial and non-spatial coupling. Spatial coupling reflects their spatial distribution characteristics, indicating a relationship of mutual enhancement or restraint, while non-spatial coupling captures various states of coordinated development between the two. For a given spatial unit, there are theoretically four types of spatial coupling relationships between development intensity and landscape pattern: high-high, high-low, low-high, and low-low. Additionally, in terms of non-spatial coupling, there exists a range of coupling coordination states that vary from disordered to harmonized. The theoretical analysis outlined above is illustrated in Fig. 1.

This study aims to uncover the spatial differentiation and coupling characteristics between spatial development intensity and landscape pattern within villages, a micro-level land-use unit. By doing so, it seeks to provide a reference for decision-making in village land development, conservation, and landscape pattern optimization. This research also addresses the current gap in micro-scale studies of spatial development intensity and landscape patterns, thus enriching the research framework for studying these spatial dynamics.

## Research methods

**Fig. 2:**
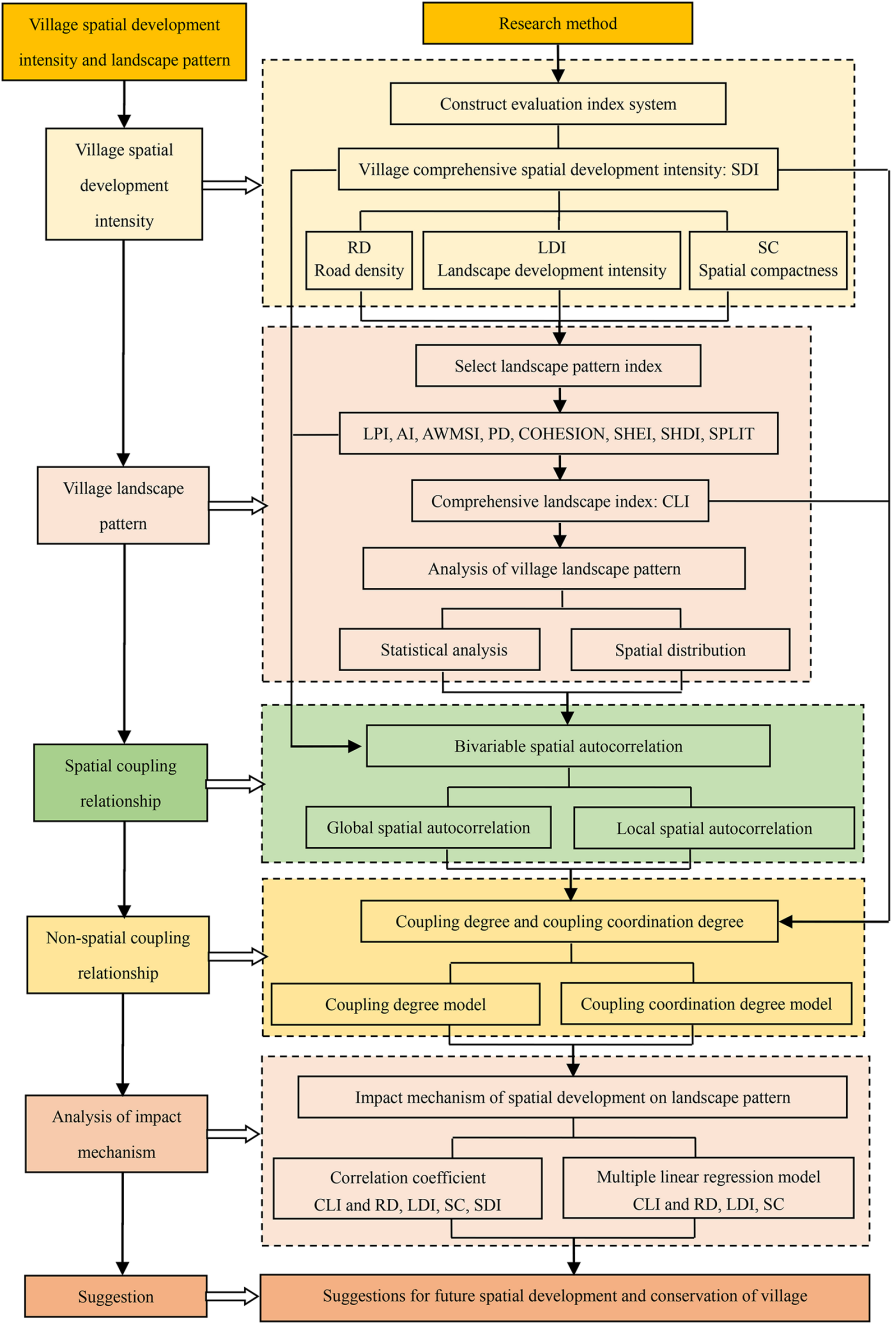
The framework diagram of the technical methodology.

The study comprises six specific methodological steps. First, the comprehensive index method is applied to calculate village spatial development intensity. Second, landscape pattern indices are utilized to analyze the characteristics of the village’s landscape pattern and to compute a comprehensive landscape index. Third, a bivariate spatial autocorrelation model is employed to analyze the spatial coupling relationship between spatial development intensity and landscape patterns, including both global and local spatial autocorrelation. Fourth, the coupling degree and coupling coordination degree models are used to investigate the non-spatial coupling relationship between the two. Fifth, correlation coefficients and multiple linear regression models are employed to explore the mechanisms through which spatial development affects landscape pattern. Finally, an analysis of the causes of spatial development intensity and landscape pattern formation is conducted, and suggestions for the development and protection of village land space are proposed from both spatial and non-spatial perspectives. A detailed description of the research methods and technical procedures is presented in Fig. 2.

### Village spatial development intensity

Village spatial development takes village land use as the core. The study focuses on three indicators: landscape development intensity (LDI), road density (RD) and spatial compactness (SC) to calculate the village’s comprehensive spatial development intensity. Spatial development intensity (SDI) of each village is a comprehensive integration of LDI, RD and SC, that is:

1 $$SDI=\sum\limits_{{i=1}}^{3} {{w_i}{x_i}}$$

In the formula, SDI is the village spatial development intensity. *x*_*i*_ and *w*_*i*_ are the standardized values and weights of LDI, RD and SC respectively. The standardized values are calculated by range standardization method, and the weights are calculated by analytic hierarchy process method. The larger the SDI value, the higher the village spatial development intensity, the greater the interference and impact of human activities on the village.

LDI reflects the energy input of human activities to the natural ecological environment^40–42^. Different land use types have different energy input. The greater the input energy, the more severe the interference to the natural ecological environment, and the greater the intensity of landscape development. The calculation formula is:

2 $$LDI=\sum\limits_{{i=1}}^{n} {\frac{{{a_i}}}{A}} \times {I_i}$$

In the formula, LDI is the village landscape development intensity. The larger the value, the higher the degree of village landscape development, the greater the impact of human activities on the natural environment pattern. *a*_*i*_ is the area of the *i*th land use type, *A* is the total area of the village, *n* is the number of land use types, and *I*_*i*_ is the landscape development intensity coefficient of the *i*th land use type. According to existing research^43^, the landscape development intensity coefficients of different land use types are shown in Table 1.

**Table 1:** The landscape development intensity coefficients of different land use types (*I*).

First-level land use type	Secondary land use type	I	First-level land use type	Secondary land use type	I
Agricultural land	Cultivated land	4.54	Unused land	Bare land, Tidal flat	1
	Abandoned land	1	Water area	River	1
	Garden land	3.68		Artificial water pond, Reservoir	1.83
Transportation land	Railway, Highway	7.81	Construction land	settlement (Rural)	6.79
Natural vegetation	Woodland	1		Residential -commercial land (Town )	8.66
	Shrubland	1		Public service facility (school, clinic, etc.)	8.07
	Grassland	1	Industrial and mining land	Manufacturing, Mining industries	8.32

Road density refers to the ratio of the total length of roads in the village to the area of the village, which is the core index to evaluate the construction of the village transportation system. As the basic structure of village space, the road is the basic support of village spatial development. Therefore, its density is also an important index to evaluate village spatial development intensity. Compactness was first used to study the urban sprawl effect in the process of urbanisation. It is a measurement method based on the concept of “compact city”^44–46^, which is mainly used to study the spatial form of the region. The closer the spatial form is to the circle, the higher the spatial compactness. The spatial compactness reflects the form of village spatial development and is a measure of the results of village spatial development. The higher the spatial compactness, the more centralised the village spatial development is. The calculation formulas of road density and spatial compactness are:

3 $$RD=L/A$$

4 $$SC=2\sqrt {\pi S} /P$$

Formula (3): RD is the road density, *L* is the length of the road, and *A* is the total area of the village. The larger the RD value, the higher the road density of the village, the better the traffic conditions, and the more conducive it is to spatial development. Formula (4): SC is the compactness, and *S* and *P* are the area and circumference of the village construction land respectively. A higher SC value indicates that the village’s construction land is more compact and centrally distributed.

To sum up, it can be seen that LDI reflects the type and degree of land use development of the village, RD reflects the ability and conditions of the village to support spatial development and construction, and SC can evaluate the form of village spatial development. LDI, RD, and SC can respectively measure village spatial development intensity from a specific aspect, and their organic integration can comprehensively evaluate village spatial development intensity.

### Landscape pattern index

Landscape pattern index is a classic method to analyse the spatial characteristics of landscape patterns, which can effectively reflect the landscape pattern information of land use^47,48^. Eight indicators, including LPI (largest patch index), AI (aggregation index), AWMSI (area weighted mean shape index), PD (patch density), COHESION (patch cohesion index), SHEI (Shannon’s evenness index), SHDI (Shannon’s diversity index) and SPLIT (splitting index), are selected to calculate village spatial pattern characteristics. LPI and AWMSI reflect the shape characteristics of landscape patches, while PD measures the density of patch distribution. AI indicates the aggregation or dispersion of the landscape pattern, and COHESION reflects the connectivity level between landscape patches. SHEI and SHDI provide information on the spatial distribution of patches, while SPLIT measures the degree of fragmentation of the landscape patches. These eight indices reveal landscape pattern characteristics from different perspectives and have significant ecological relevance. Theoretically, larger LPI and AWMSI values indicate that the ecological environment retains more of its original natural landscape pattern and is less disturbed by human activities. In such cases, the values of the other six indices tend to be relatively lower. Conversely, smaller LPI and AWMSI values suggest greater disturbance to the ecological environment from human activities, with the natural landscape pattern becoming more artificial. Under these conditions, the values of the other six indices are relatively higher. The calculation formula and ecological significance of the eight landscape pattern indexes can be found in the relevant literature^49^, which will not be repeated here.

The above eight landscape pattern indexes reflect the landscape pattern characteristics of the village from a specific aspect, and the integration of them will obtain the village comprehensive landscape index (CLI). CLI comprehensively reflects the degree of interference of human activities on the original natural landscape of the village. The greater the interference of human activities, the greater the comprehensive fragmentation of the village landscape, and vice versa. The calculation formula is as follows:

5 $$CLI=\sum\limits_{{i=1}}^{8} {{w_i}{x_i}}$$

In the formula, CLI is the comprehensive landscape index of the village. *x*_*i*_ and *w*_*i*_ are the standardized values and weights of the eight landscape pattern indexes respectively. The standardized values are calculated by range standardization method, and the weights are calculated by the entropy method. Among the eight landscape pattern indices, LPI and AWMSI are negative indicators. The larger the value, the greater the village can maintain the original natural landscape and the less interference of human activities. The remaining six indexes are positive indicators. The larger the value, the greater the interference and impact of human activities on the village.

### Bivariate spatial autocorrelation

Spatial autocorrelation is a measure of the degree of attribute value aggregation of a space element, which aims to measure whether the attribute value of a certain point in space is correlated with its adjacent point^50^. Bivariate spatial autocorrelation is the deepening of traditional univariate spatial autocorrelation, which can reveal the spatial correlation between multiple variables, that is, the correlation between the attribute value of the space element and other attribute values in adjacent space. Spatial autocorrelation includes global spatial autocorrelation and local spatial autocorrelation. Global spatial autocorrelation can generally reflect the similarity between each space unit and the adjacent space unit. It is often measured by global Moran’s I. Local spatial autocorrelation can reflect the aggregation and differentiation of local space elements. Local Moran’s I is commonly used. The calculation formulas are:

6 $$I=\frac{{\sum\limits_{{i=1}}^{n} {\sum\limits_{{j=1}}^{n} {{w_{ij}}({x_i} - \overline {x} )({x_j} - \overline {x} )} } }}{{{S^2}\sum\limits_{{i=1}}^{n} {\sum\limits_{{j=1}}^{n} {{w_{ij}}} } }}$$

7 $${I_i}=\frac{{({x_i} - \overline {x} )}}{{{S^2}}}\sum\limits_{{i,j=1}}^{n} {{w_{ij}}({x_j} - \overline {x} )}$$

In the formula, *I* is the global Moran’s I, and *I*_*i*_ is the local Moran’s I of unit *i*. *n* is the number of spatial units, and *x*_i_ and *x*_*j*_ are the attribute values of units *i* and *j* respectively; *w*_*ij*_ is a spatial weight based on spatial adjacency relationship. The *w* = 1 when *i* and *j* are adjacent to each other, or else, they are 0. The range of global Moran’s I is (-1, 1). When the value is greater than 0, it is spatially positively correlated, which means that the spatial agglomeration distribution; when the value is less than 0, it is spatially negatively correlated, which means that the spatially distributed. When the value is 0, there is no spatial correlation. At this time, the spatial distribution is randomly distributed. The LISA distribution map is usually based on the Local Indicators of Spatial Association (LISA), which is used to show the high-high (H-H), low-low (L-L) spatial positive correlation between the research index value and the average of its neighborhood indicator value, or the high-low (H-L), low-high (L-H) spatial negative correlation and the absence of significant spatial randomness.

### Coupling degree and coupling coordination degree

Spatial autocorrelation can reveal the spatial coupling relationship and characteristics of village spatial development intensity and landscape pattern, but it cannot reveal the non-spatial coupling relationship between them. Therefore, the coupling degree and coupling coordination degree model are used to analyse the non-spatial coupling relationship between village spatial development intensity and landscape pattern. The concept of coupling degree originates from the capacity coupling coefficient model in the field of physics and has since been widely adopted in social sciences. Coupling degree is used to describe the strength of interaction and influence between two or more systems. Building upon this concept, the coupling coordination degree was developed to indicate whether these interactions are positive or negative. A positive interaction promotes system development, reflecting coordinated development, while a negative interaction hinders system development, indicating disordered development. This metric enables the evaluation of the transition and trend between disordered and uncoordinated states to coordinated and orderly states among systems. Human society is a complex system where multifaceted coupling relationships exist among economic, social, and environmental subsystems. To evaluate such relationships, various models have been developed, including the environmental Kuznets curve, system dynamics model, grey relational model, spatial regression model, and coupling degree and coupling coordination degree model. Among these, the coupling degree and coupling coordination degree model serves as an effective analytical tool. Compared to other models, it is easier to understand and apply, features a relatively straightforward calculation process, and provides more intuitive results. A higher coupling degree indicates stronger interactions between systems, while a lower degree suggests weaker interactions. Similarly, a higher coupling coordination degree reflects a trend toward coordinated development between systems, whereas a lower degree indicates a tendency toward disordered development. Due to these distinctive characteristics, this model has been widely applied in empirical studies examining the coupling relationships between economic, social, and environmental systems across various scales and regions. In this study, the coupling degree can reflect the intensity of interaction and influence between village spatial development intensity and landscape pattern, and the coupling coordination degree can reveal whether the interaction and influence are coordinated and the level of coordinated development. The calculation methods are as follows:

8 $$C=\frac{{2\sqrt {{U_1}{U_2}} }}{{{U_1}+{U_{}}}}$$

9 $$T=\sum\limits_{{i=1}}^{2} {{a_i}{U_i}}$$

10 $$D=\sqrt {C \times T}$$

In the formula, *C* is the coupling degree between village spatial development intensity and landscape pattern; *U* is the village spatial development intensity (SDI) and the comprehensive landscape index (CLI); *T* is the system comprehensive coordination index; and *D* is the coupling coordination degree index. SDI and CLI are both indispensable parts of the village development system, so the contribution *a* of SDI and CLI is assigned to 0.5 when calculating *T*. Referring to the relevant research^51,52^, the coupling degree is divided into 5 levels, corresponding to 5 coupling types, and the coupling coordination degree is divided into 10 levels, corresponding to 10 coupling coordination types (Table 2).

**Table 2:** Coupling types and coupling coordination types.

Coupling degree	Coupling types	Coupling coordination degree	Coupling coordination types
0 < D ≤ 0.2	Uncoupling	0 < D ≤ 0.1	Extreme disordination
		0.1 < D ≤ 0.2	Severe disordination
0.2 < D ≤ 0.4	Less coupling	0.2 < D ≤ 0.3	Intermediate disordination
		0.3 < D ≤ 0.4	Light disordination
0.4 < D ≤ 0.6	Low coupling	0.4 < D ≤ 0.5	Critical disordination
		0.5 < D ≤ 0.6	Barely ordination
0.6 < D ≤ 0.8	Medium coupling	0.6 < D ≤ 0.7	Primary ordination
		0.7 < D ≤ 0.8	Intermediate ordination
0.8 < D ≤ 1.0	High coupling	0.8 < D ≤ 0.9	Good ordination
		0.9 < D ≤ 0.10	High quality ordination

## Case study

### Research area and data

#### Research area

**Fig. 3:**
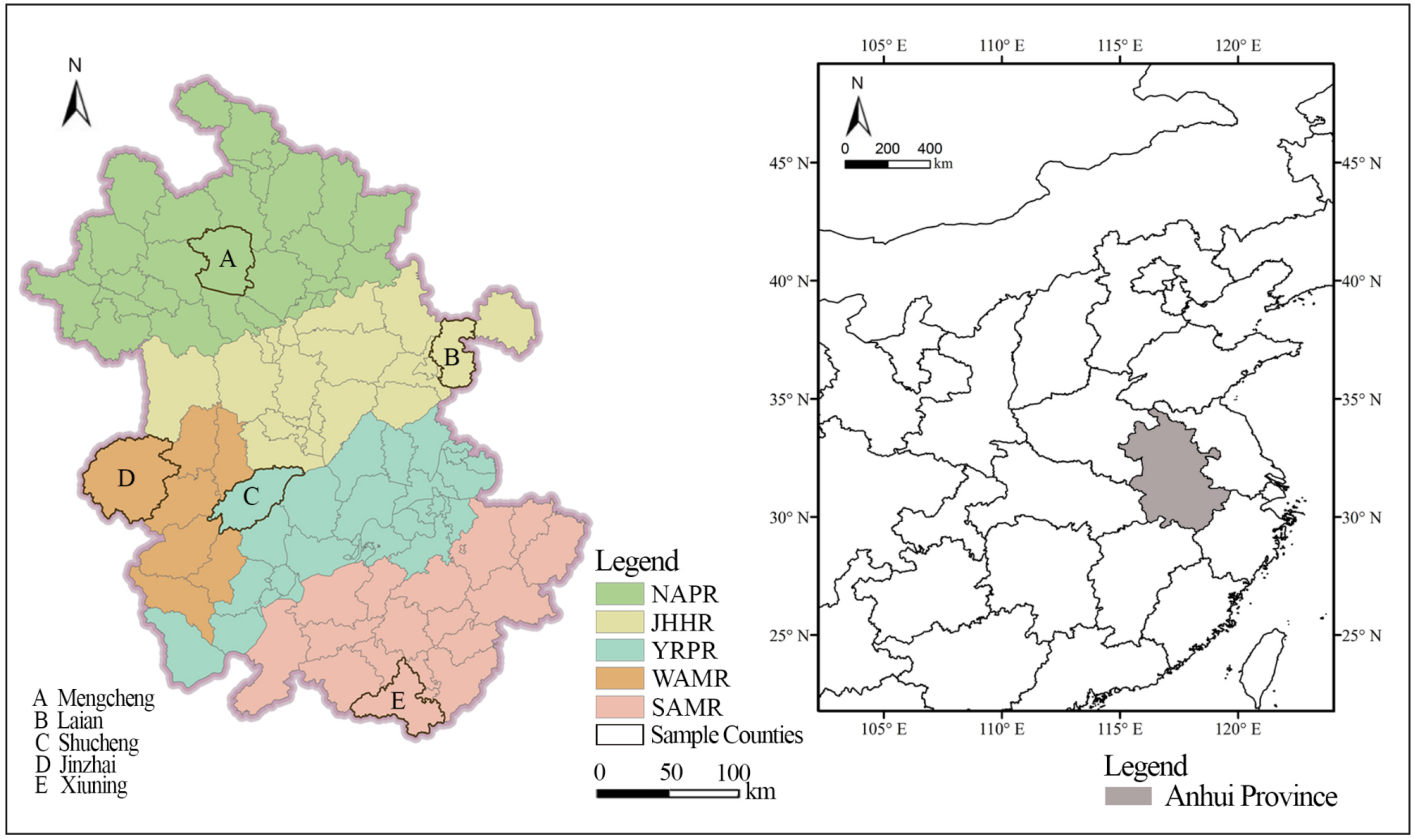
Location map of the study area. The process of creating Fig. 3 was as follows: First, the location map of Anhui Province within China and the administrative division map of Anhui Province were drawn in ArcGIS 10.7 and exported as two separate images. These images were then arranged and compiled in Adobe Photoshop CS6 to produce Fig. 3.

 Anhui Province is located in the eastern part of mainland China, situated in the heart of the Yangtze River Delta, between 114°-120° E longitude and 29°-35° N latitude (Fig. 3). It is an important part of the two national development strategies of the Yangtze River Economic Belt and the integrated development of the Yangtze River Delta, with a total area of about 141,000 km^2^. Anhui Province has a total of 16 prefecture-level cities and 61 counties, with a permanent population of 61.27 million people. Of this population, 60.15% resides in urban areas, while 39.85% resides in rural areas. Anhui Province is famous for its rich and diverse terrain and landforms, including plains, hills, mountains, rivers, and lakes. The world famous Yangtze River flows through Anhui Province and the famous Huangshan Mountain is located in Anhui Province. Overall, Anhui Province includes five geographical regions: northern Anhui plain region (NAPR), along the Yangtze River plain region (YRPR), Jiang-huai hilly region (JHHR), southern Anhui mountainous region (SAMR), western Anhui mountainous region (WAMR). There are large regional differences among the five geographical regions.

NAPR is the largest and most densely populated area in Anhui Province. Benefiting from its flat terrain and fertile land, this region is Anhui’s most crucial agricultural area and a significant grain-producing area in China. The Yangtze River plain, located along both banks of the Yangtze River, features relatively flat terrain interspersed with mountains, rivers, lakes, and ridges, making it another key grain-producing area in Anhui. The Jiang-huai Hills, situated in central Anhui, consist of terraces, hills, and river valleys, creating a varied terrain that fosters unique natural landscapes and ecological environments. SAMR is predominantly mountainous, with many mountain ranges, making it one of China’s key mountain-concentrated areas and an essential national ecological function zone. WAMR is primarily composed of the Dabie Mountains, with complex and diverse terrain that includes high mountains, hills, and valleys, marking it as an important ecological function zone in Anhui Province.

Different natural geographical environments bring different basic conditions to the village spatial development in Anhui Province, which in turn have an important impact on the village landscape pattern. Starting from the perspective of typicality and representativeness, 20 villages in one county in each geographical regions of Anhui Province are selected as samples, with a total of 100 sample villages. The details are shown in Fig. 3; Table 3. The 100 sample villages represent the five geographical regions of Anhui Province, which can reveal the spatial distribution characteristics and coupling relationship of village spatial development intensity and landscape pattern of Anhui Province as a whole.

**Table 3:** List of sample villages.

Region (county)	Village
NAPR (Mengcheng county)	Baimiao village, Chaihe village, Chitang village, Cibei village, Daliji village, Dingtang village, Erlang village, Fengguang village, Geqiao village, Humiao village
	Huanglong village, Laoying village, Lidazhai village, Luwafang village, Mamiao village, Qumiao village, Sanguan village, Shuanglong village, Taiping village, Xuehu village
JHHR (Laian county)	Banta village, Baoshan village, Beijian village, Chenguan village, Daliuying village, Dayuying village, Doushan village, Gaochang village, Gaoying village, Hongqi village
	Hongxing Village, Jingbo Village, Leiguan Village, Wangji Village, Wuli Village, Xinggang Village, Yanchen Village, Yangdu Village, Yuzhuang Village, Yutang Village
YRPR (Shucheng county)	Dengtang Village, Fengxi Village, Vangyao Village, Guanguou Village, Wan Village, Hanwan Village, Hantang Village, Huanggang Village, Kongyan village, Masong Village
	Wutang Village, Qimenyan Village, Quanyan Village, Shunhe Village, Tangshu Village, Wuyang Village, Xiweir Village, Yucheng Village, Yuantang Village and Yuejin Village
WAMR (Jinzhai county)	Chawang Village, Chuanchong village, Dadukou Village, Donggao Village, Gongdian Village, Guanmiao Village, Guangming Village, Guodian Village, Huashi Village, Huangyan Village
	Jiushu Village, Moyuan Village, Qiaodian Village, Qingshui Village, Sanhe Village, Taiping Mountain Village, Xuchong Village, Rouge Village, Changyuan Village, Zhu Mountain Village
SAMR (Xiuning county)	Fanggan Village, Fangkou village, Gaotan village, He village, He Keng village, Huaqiao village, Huangcun village, Huangyuan village, Jixi village, Jinzhu village
	Qiaokeng Village, Sancun Village, Ishida Village, Shuangqiao Village, Xikou Village, Yanli Village, Yangzhuang Village, Yixin Village, Yuankou Village, Zhonghe Village

#### Data source

The research data mainly include administrative division data, remote sensing imaging data and road traffic data of 100 villages. Administrative division data and remote sensing imaging data were sourced from the Data Center for Resources and Environmental Sciences of the Chinese Academy of Sciences (https://www.resdc.cn), while road traffic data were obtained from Open Street Map (https://www.openstreetmap.org) and the National Catalogue Service for Geographic Information (https://www.webmap.cn). Human-computer interactive interpretation of remote sensing images of 100 villages is performed on the ArcGIS platform. The study divides village land use into cultivated land, garden land, woodland, grassland, roads, rivers, pond water surfaces, reservoirs, rural settlements, etc., so as to obtain village land use vector data. In ArcGIS, a spatial topology check was performed on the road data, combined with manual verification, to ensure that no duplicate lines existed in the road data. This process resulted in a complete road network dataset for all villages. The spatial scope of all villages is divided into 50 m×50 m grids as the basic spatial unit for computational analysis.

### Spatial differentiation of village development intensity and landscape pattern

#### Analysis of village spatial development intensity

The calculation results of the spatial development intensity of 100 villages in Anhui Province are shown in Table 4. In terms of spatial compactness, the average value of villages in YRPR is the highest of 0.0399, followed by 0.0369 in SAMR, and the average values of NAPR, JHHR and WAMR are very similar. Regarding road density, the average value of villages in NAPR is the highest of 6.7318 km/km^2^, followed by JHHR, YRPR and WAMR, and the lowest average value is only 2.6697 km/km^2^ which is in SAMR. As for landscape development intensity, the average value of villages in NAPR is the highest at 4.6321, followed by YRPR and JHHR. The average values in SAMR and WAMR are similar, and they are significantly smaller than those of the other three sub-regions. In terms of comprehensive spatial development intensity, the average value of villages in NAPR is the highest of 0.5443, slightly higher than 0.5156 in YRPR, followed by 0.4129 in JHHR and 0.2421 in SAMR. The average value in WAMR is the lowest of only 0.1939. The village spatial development intensity in SAMR and WAMR is significantly lower than that of other sub-regions, indicating that there are significant regional differences in Anhui Province, and showing the general characteristics of “high in plain areas, medium in hilly areas, and low in mountainous areas”.

**Table 4:** Statistics of village spatial development intensity.

Region (county)	Index	SC	RD	LDI	SDI
NAPR (Mengcheng county)	Average value	0.0237	6.7318	4.6321	0.5443
	Standard deviation	0.0046	1.3660	0.1308	0.0435
JHHR (Laian county)	Average value	0.0239	5.3234	3.7129	0.4129
	Standard deviation	0.0066	1.3479	0.8284	0.1072
YRPR (Shucheng county)	Average value	0.0399	5.2122	4.2106	0.5156
	Standard deviation	0.0099	1.2360	0.8528	0.1255
WAMR (Jinzhai county)	Average value	0.0222	3.1505	2.1993	0.1939
	Standard deviation	0.0034	1.4345	0.8245	0.1206
SAMR (Xiuning county)	Average value	0.0369	2.6697	2.3082	0.2421
	Standard deviation	0.0122	1.4822	1.0037	0.1493
Total	Average value	0.0293	4.6175	3.4126	0.3818
	Standard deviation	0.0109	2.0231	1.2607	0.1813

The analysis of internal differences among five geographical regions show that the standard deviations of spatial compactness, road density and landscape development intensity of villages in SAMR are the highest, indicating that there are significant differences in spatial development between villages. The standard deviation of spatial compactness of villages in WAMR is the minimum value of 0.0034, the standard deviation of road density of villages in YRPR is the minimum value of 1.2360, and the standard deviation of landscape development intensity of villages in NAPR is the minimum value of 0.1308. In terms of the comprehensive spatial development intensity, the standard deviation of villages in SAMR is the maximum value of 0.1493, followed by YRPR, WAMR and JHHR. The standard deviation of villages in NAPR is the minimum value of only 0.0435, indicating that the village in SAMR has the maximun difference of spatial development intensity, while the village spatial development intensity in NAPR is not only the highest, but also the internal difference is the lowest. The overall average of the comprehensive spatial development intensity of 100 villages is 0.3818, and the overall standard deviation is 0.1813, indicating that the spatial development intensity of villages in Anhui Province is generally low. Meanwhile, the overall standard deviation is much greater than the internal standard deviation of the five sub-divisions. It can be seen that there is a more significant difference in the overall village spatial development intensity in Anhui Province.

**Fig. 4:**
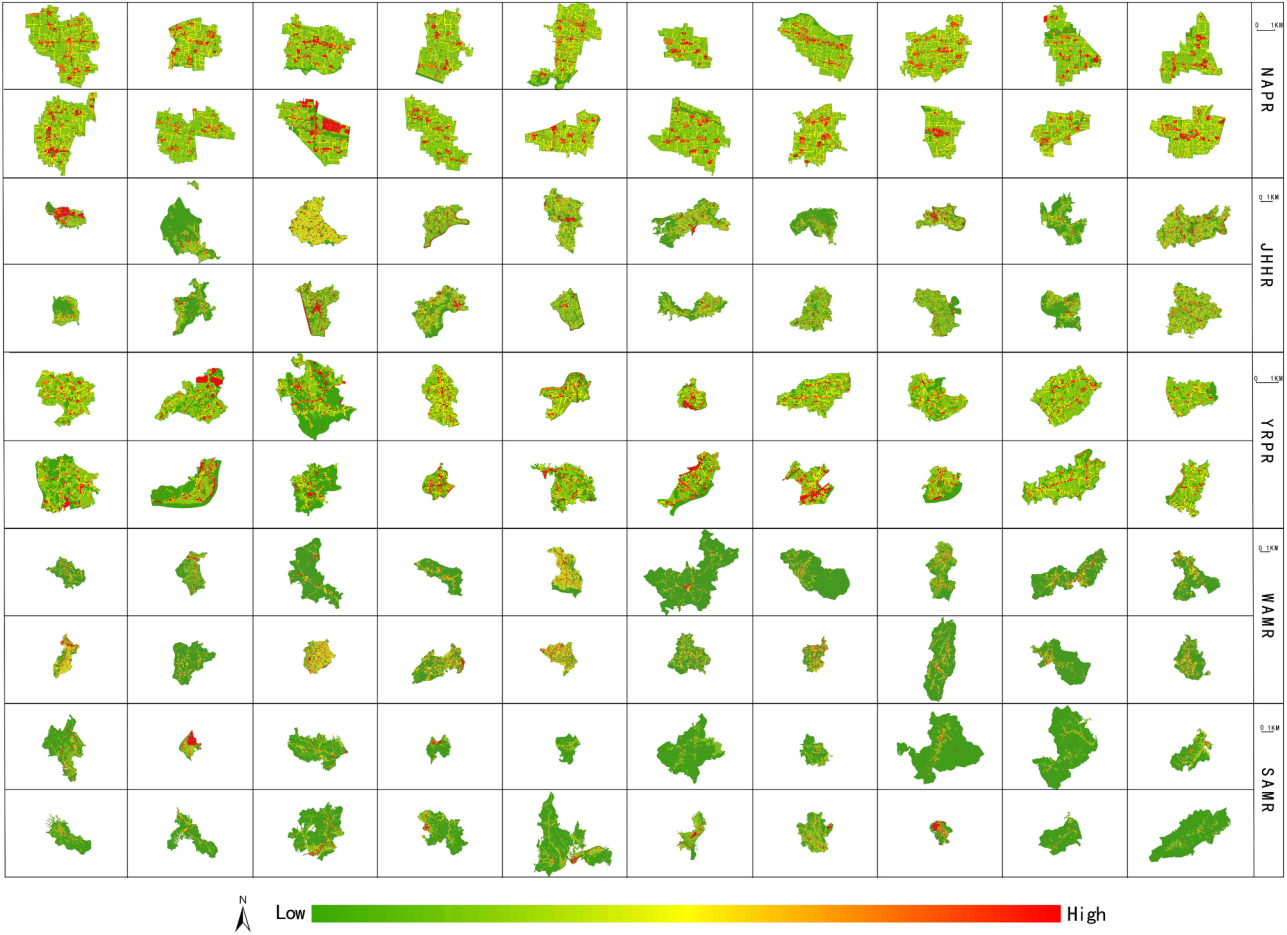
Spatial distribution map of village spatial development intensity. The process of creating Fig. 4 was as follows: First, the spatial development intensity of each village was calculated in ArcGIS 10.7, and the results were exported as separate images. These 100 images, corresponding to 100 villages, were then arranged and compiled in Adobe Photoshop CS6 to produce Fig. 4. Figures 5, 6, 7, and 8 were created using the same method as Fig. 4. (The village names in each row are consistent with those in Table 3. The following figures are the same).

The spatial development intensity of each grid in each village is calculated using the grid method, and then the spatial development intensity is divided into three levels: high, medium and low according to the equal division method. The spatial distribution map and spatial statistics of village spatial development intensity can be seen in Fig. 4; Table 5. In Fig. 4, regions with deeper red shades indicate higher spatial development intensity values, while those with greener shades reflect lower spatial development intensity values. Following this color rule, it is evident that there are significant spatial distribution differences in village development intensity across Anhui Province. There are many high-value areas of village spatial development intensity in NAPR, and showing an obvious high-value agglomeration characteristic. The spatial distribution characteristics of village spatial development intensity in JHHR and YRPR are similar, but the spatial distribution range of the high-value area in JHHR is wider than that of in YRPR. At the same time, the high-value areas in JHHR and YRPR are obviously less than that of in NAPR. The spatial distribution characteristics of village spatial development intensity in WAMR and SAMR are similar, and both have fewer high-value areas. Every village in NAPR forms distinct spatial clusters of high-value areas. There are many villages in JHHR and YRPR (such as Banta Village, Leiguan Village, Gaochang Village, Fengxi Village, Xidang Village, etc.) form high-value agglomeration areas. Only three villages in SAMR (Fangkou Village, Xikou Village, Yixin Village) form high-value agglomeration areas, while villages in WAMR have not formed obvious high-value agglomeration areas.

**Table 5:** Spatial zone statistics of village spatial development intensity.

Region (County)	The total number of grids	Level	The number of grids	Proportion (%)
NAPR (Mengcheng county)	68,811	High	7940	11.54
		Medium	55,283	80.34
		Low	5588	8.12
JHHR (Laian county)	70,734	High	5661	8.00
		Medium	51,986	73.50
		Low	13,087	18.50
YRPR (Shucheng county)	26,051	High	2998	11.51
		Medium	19,339	74.24
		Low	3714	14.26
WAMR (Jinzhai county)	133,198	High	3103	2.33
		Medium	32,279	24.23
		Low	97,817	73.44
SAMR (Xiuning county)	26,966	High	2881	10.68
		Medium	19,690	73.02
		Low	4395	16.30

According to Table 5, the maximum number of high-value grids of village spatial development intensity in NAPR is 7940, accounting for 11.54% of the total number of village grids s in NAPR. The number of high-value grids in JHHR is 5661, and the number of high-value grids in YRPR and WAMR is basically the same. The minimum number of high-value grids in SAMR is 2881. Except for WAMR, the other four sub-regions have the largest number of median grids, accounting for more than 70%, which is in an absolute dominant position. Except for NAPR, the number of low-value grids in the other four sub-regions is greater than the number of high-value grids. The number of low-value grids in WAMR is as high as 97,817, far exceeding that of other regions. Besides, the proportion of the sum of the number of median and low value grids in WAMR is as high as 97.67%, which is obviously different from the dominance of the number of high and median value grids in other sub-regions. This once again shows that villages in WAMR have the lowest spatial development intensity. Generally speaking, the number of high and low value grids of village spatial development intensity in Anhui Province is relatively small, and the number of median grids is the largest, showing a typical normal distribution characteristic.

#### Analysis of village landscape pattern

The calculation results of landscape pattern index of 100 villages in Anhui Province are shown in Table 6. According to Table 6, the village landscape pattern in the five regions is obviously different. Taking LPI as an example, the average value of villages in JHHR is the highest of 34.7137, followed by SAMR, WAMR and YRPR. The average value of villages in NAPR is the lowest of only 3.3556, about 10% of villages in JHHR. Obviously, there are significant regional differences. Furthermore, the standard deviation reveals considerable differences among the various regions. The villages in JHHR has the maximum standard deviation of 18.2536, while the standard deviation of villages in NAPR has the minimum value, which is only 1.6512. The difference between them is about 11 times. Based on the eight landscape pattern indexes, it can be seen that the village landscape patterns of the five regions in Anhui Province show different characteristics.

**Table 6:** Statistics of village landscape pattern indexes.

Region (county)	Index	LPI	AI	AWMSI	PD	COHESION	SHEI	SHDI	SPLIT
NAPR (Mengcheng county)	Average value	3.3556	85.4837	1.2188	64.9994	70.5817	0.8780	5.4375	143.5007
	Standard deviation	1.6512	2.5568	0.0942	5.3677	2.1246	0.0183	0.2693	43.4302
JHHR (Laian county)	Average value	34.7137	64.9960	4.6427	44.0597	92.2353	0.5661	1.7592	12.2443
	Standard deviation	18.2536	4.7084	1.9197	8.7964	3.3029	0.0684	0.2509	12.1955
YRPR (Shucheng county)	Average value	6.9803	69.9944	1.5205	92.1717	68.0840	0.8529	4.7985	75.8322
	Standard deviation	4.9166	4.5944	0.2008	21.6492	7.9840	0.0531	0.4393	41.2270
WAMR (Jinzhai county)	Average value	18.2462	76.3977	2.5269	54.4669	86.3315	0.6467	4.1614	30.4251
	Standard deviation	13.0307	8.7686	0.4785	24.6758	7.2465	0.1387	0.9749	35.4832
SAMR (Xiuning county)	Average value	19.4188	73.3615	2.6206	62.0378	83.8432	0.6853	4.1539	27.6621
	Standard deviation	11.5477	6.3540	1.0396	24.2118	8.5454	0.1075	0.7881	34.4265

The average value of LPI of villages in NAPR is the lowest, while the average value of SPLIT is the highest, indicating a high intensity of human activities and a high degree of landscape fragmentation. The average values of AI, SHEI and SHDI are high, and the aggregation, uniformity and diversity are high; the average values of AWMSI, PD and COHESION are low, indicating that the land patches are more regular in shape, the heterogeneity and fragmentation of landscape are high, and the spatial connectivity of the landscape is low. In general, the village landscape pattern in NAPR is mainly characterised by high aggregation and high fragmentation, which means that village in NAPR is the most affected by human activities. It can also be seen that the village landscape pattern in JHHR has less fragmented and aggregation, smaller heterogeneity, and higher connectivity, which means that village in JHHR is less affected by human activities. The village landscape pattern in YRPR has high heterogeneity and fragmentation, and low agglomeration, which means that village in YRPR is greatly affected by human activities. The village landscape pattern indexes of SAMR and WAMR are similar. The landscape is relatively aggregated and has low fragmentation. Meanwhile, the landscape shape is relatively complex, the landscape connectivity is relatively high, but the heterogeneity is relatively small, which means that villages in SAMR and WAMR are less affected by human activities.

**Fig. 5:**
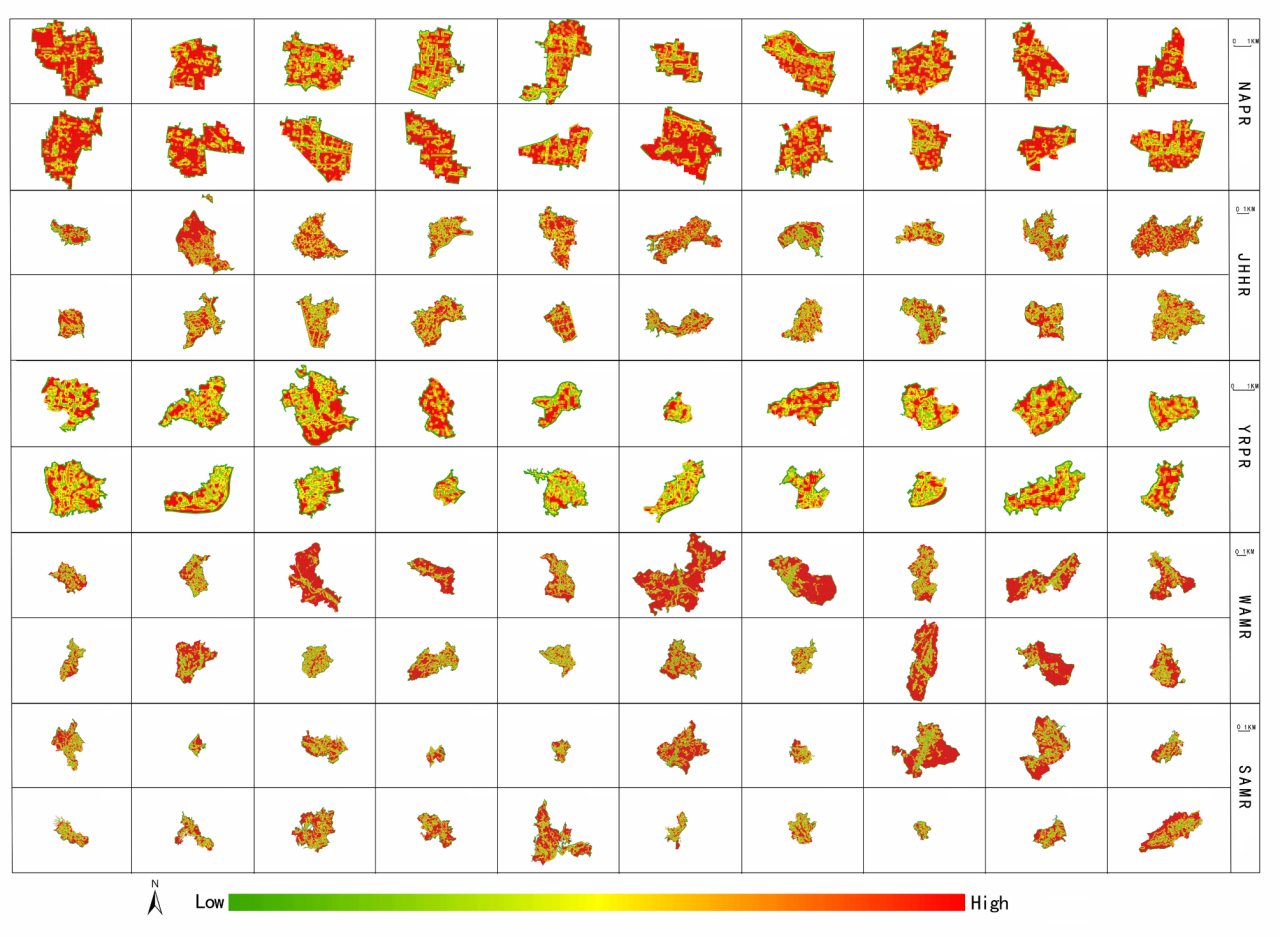
Spatial distribution map of LPI.

**Fig. 6:**
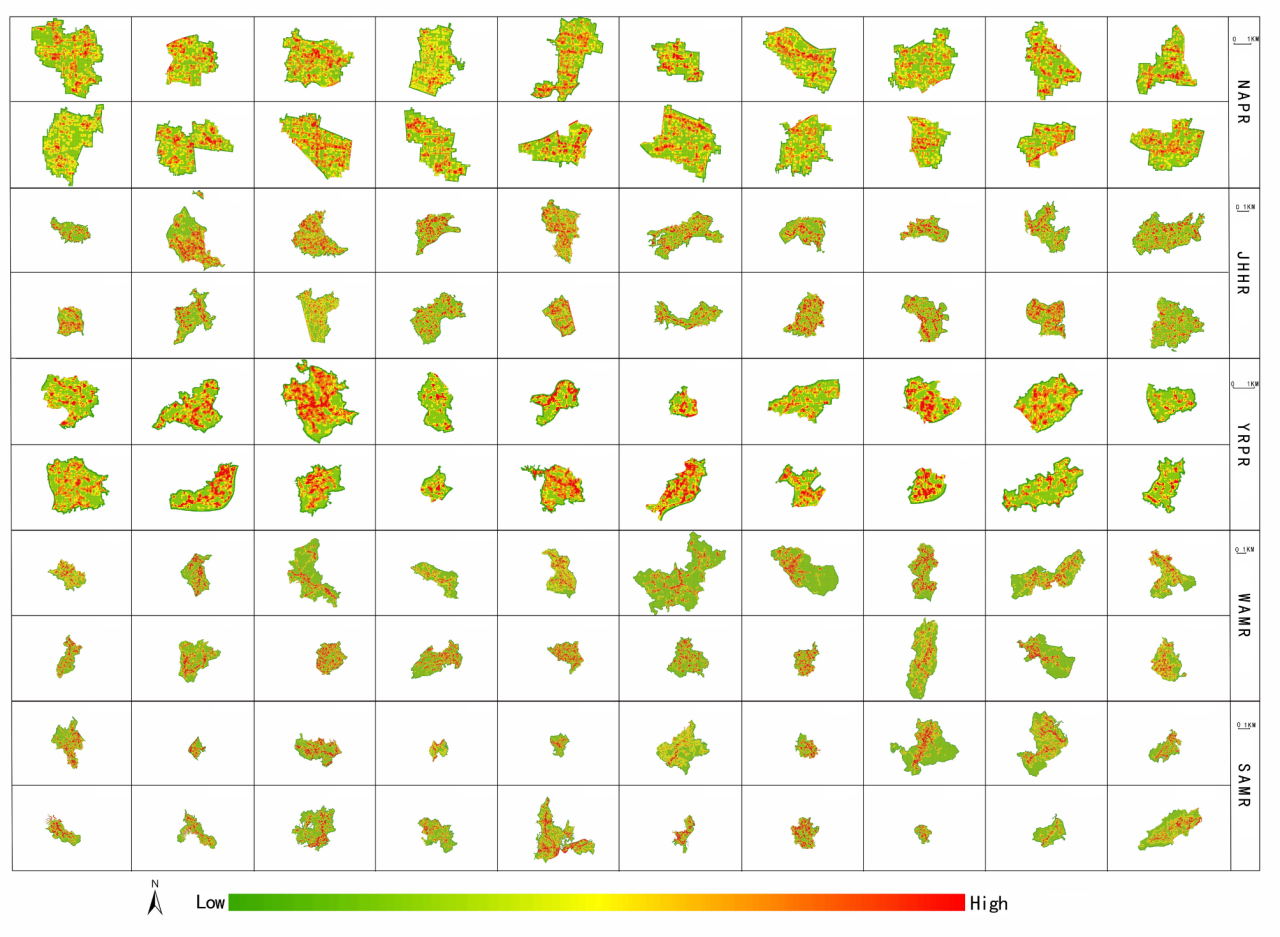
Spatial distribution map of PD.

The landscape pattern index of each grid in each village is calculated using the grid method, and the spatial distribution map of the village landscape pattern index is obtained. Overall, the spatial distribution pattern of LPI of all villages is basically the same (Fig. 5), and the spatial distribution of AI and COHESION is similar to that of LPI. The spatial distribution pattern of PD of all villages is basically the same (Fig. 6), and the spatial distribution of SHDI and SPLIT is similar to that of PD and presents a spatial distribution trend contrary to the high and low values of LPI, AI and COHESION. The AWMSI and SHEI do not show obvious spatial distribution characteristics.

In Fig. 5, areas shaded in deeper red indicate higher LPI values, while those in greener shades have lower LPI values. Similarly, in Fig. 6, as the color transitions from green to red, the PD values increase. Following this color rule, it can be observed that there is a certain degree of similarity in the spatial distribution of village landscape patterns across Anhui Province. According to Figs. 5 and 6, the high-value areas of LPI, AI and COHESION of villages in NAPR are the most widely distributed, mainly concentrated in cultivated land areas, and the low-value areas are mainly distributed around village settlements and roads and other construction land. The high-value areas of PD, SHDI and SPLIT are mainly distributed in the village construction land area, and the low-value areas are mainly concentrated in the cultivated land area. The distribution of low-value areas of LPI, AI, COHESION and high-value areas of PD, SHDI and SPLIT in JHHR are both less and presente as scattered network structures, while the high-value areas of the former and the low-value areas of the latter are concentrated in the areas between the network structures. The high-value areas of LPI, AI, COHESION, PD, SHDI and SPLIT of villages in YRPR are scattered, and the low-value areas are relatively less. The high-value areas of LPI, AI, COHESION and low-value areas of PD, SHDI, SPLIT are mainly distributed outside of the villages, while the low-value areas of the former and the high-value areas of the latter have centre points and scatter around or to one side from the centre points. Combined with the village land use data, it can be seen that the high-value areas of LPI, AI and COHESION are mainly distributed in cultivated land and woodland areas, while the low-value areas are distributed in village settlements, roads and other construction land areas. The high-value areas of PD, SHDI and SPLIT are mainly distributed in the village construction land area, and the low-value areas are mainly distributed in the cultivated land and woodland areas. Based on the above analysis, it can be seen that LPI, AI, COHESION and PD, SHDI, SPLIT form a spatial distribution pattern in which the main distribution areas of high-value and low-value are opposite.

### Coupling relationship between village development intensity and landscape pattern

#### Spatial coupling characteristics of village spatial intensity and landscape pattern

The overall spatial coupling characteristics between village spatial development intensity (SDI) and landscape pattern (LP) are analysed using bivariate global spatial autocorrelation. The results are shown in Table 7. The global Moran’s I of village spatial development intensity and eight landscape pattern indexes in Anhui Province is all greater than 0. Meanwhile, they have all passed the significance test with a p-value of 0.01, indicating that there is a significant spatial positive correlation between village spatial development intensity and landscape pattern, and both exhibit a spatial pattern of clustering. Furthermore, local spatial autocorrelation analysis is used to identify the specific spatial coupling characteristics of spatial development intensity and landscape pattern, including four clustering patterns: high-high, low-low, high-low and low-high.

**Table 7:** Global spatial autocorrelation of village spatial development intensity and landscape pattern.

SDI-LP	Moran’s I	SDI-LP	Moran’s I
SDI-LPI	0.126	SDI-COHESION	0.127
SDI-AI	0.129	SDI-SHEI	0.140
SDI-AWMSI	0.140	SDI-SHDI	0.140
SDI-PD	0.178	SDI-SPLIT	0.140

**Fig. 7:**
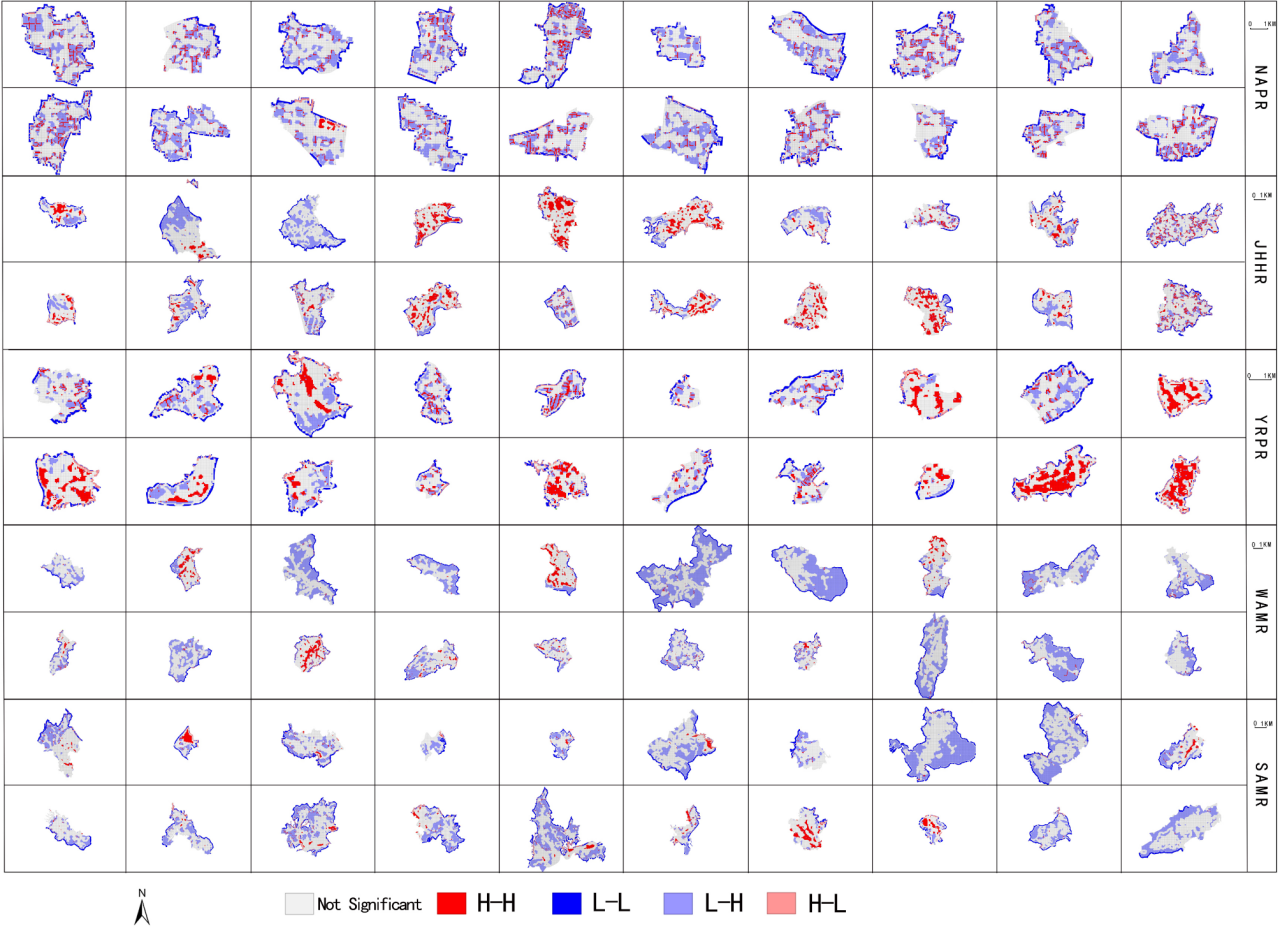
Spatial coupling distribution map of village spatial development intensity and LPI.

In general, the spatial clustering characteristics of village spatial development intensity and LPI are pronounced (Fig. 7). In Fig. 7, the four colors represent four significant spatial coupling relationships between village spatial development intensity and LPI: high-high (H-H) clustering, low-low (L-L) clustering, low-high (L-H) and high-low (H-L) clustering, with gray indicating non-significant spatial coupling relationships. Based on this, it can be understood that high-high and low-high clustering are the dominant coupling types. The spatial coupling characteristics of AI and COHESION are similar to LPI. Villages in YRPR have the largest number of high-high clustering, showing a typical cluster and block distribution. The number of high-high clustering in JHHR and NAPR is significantly less than that in JHHR. The number of high-high clustering in WAMR and SAMR is the least. Except for some villages with certain high-high clustering, other villages are scatteredly distributed in small quantities. However, there are a large number of low-high clustering in WAMR and SAMR, which are also showing a typical cluster and block distribution. Meanwhile, the low-high clustering in other sub-regions is not obvious. Combined with the village land use data, it is found that the high-high clustering of villages in five regions is mainly distributed in cultivated land and construction land such as homesteads and industrial land. Especially the villages in YRPR, their high-high clustering are concentrated in large areas of cultivated land and construction land, forming a clear spatial clustering distribution trend. Due to the limitations of terrain and landforms, both cultivated land and construction land are limited and dispersed in WAMR and SAMR, resulting in sporadic distribution of high-high clustering. Low-high clustering is mainly concentrated in woodland, especially the villages in WAMR and SAMR, their woodland resources are abundant and widely distributed, thus forming an obvious low-high clustering distribution pattern.

**Fig. 8:**
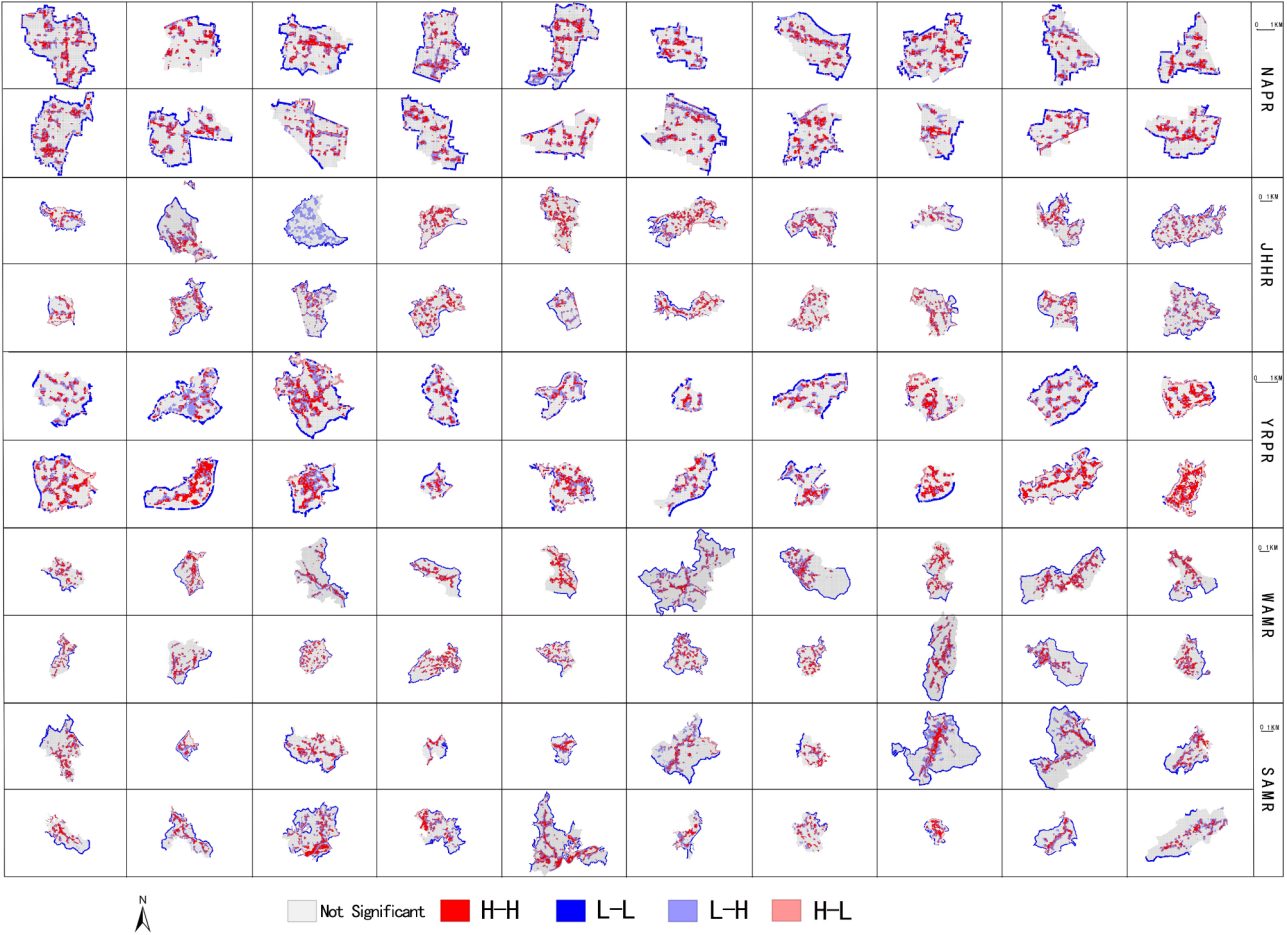
Spatial coupling distribution map of village spatial development intensity and PD.

The spatial clustering characteristics of village spatial development intensity and PD are also pronounced (Fig. 8). In Fig. 8, the four colors represent four significant spatial coupling relationships between village spatial development intensity and PD: high-high clustering, low-low clustering, low-high clustering, and high-low clustering, with gray indicating non-significant spatial coupling relationships. Based on this, it can be inferred that high-high clustering is the dominant coupling type. The spatial coupling characteristics of SHEI, SHDI and SPLIT are similar to PD. The high-high clustering of all villages is relatively obvious, and showing a typical band, cluster and block distribution. The high-high clustering of villages in YRPR is the most obvious, and some villages show a large-scale clustering distribution trend. There are also certain low-low clustering of villages in JHHR, WAMR and SAMR, but they are relatively few and not prominent. Combined with the village land use data, it can be seen that high-high clustering are mainly distributed in cultivated land and construction land areas, and low-high clustering are mainly concentrated in fragmented woodland areas around high-high clustering. In addition, the village spatial development intensity and AWMSI form a coupling type dominated by high-high clustering. Among them, the high-high clustering of villages in YRPR is relatively prominent, showing a more obvious clustering distribution trend. However, high-high clustering has no significant distribution characteristic of land types, which are mainly concentrated in fragmented patch areas of various land types.

Further, the spatial coupling relationship between spatial development intensity (SDI) and landscape pattern of all villages can be summarised into two categories. One type is SDI and LPI, AI, COHESION, and the other type is SDI and PD SHEI、SHDI、SPLIT. The coupling relationship in each category has similar spatial distribution characteristics and includes high-high and low-high clustering, as detailed in Table 8. The high-high clustering of SDI and LPI, AI and COHESION corresponds to fragmented settlements, roads, various large areas of development and construction land and cultivated land, and low-high clustering corresponds to woodland, mountains and water bodies. High-high clustering of SDI and PD, SHEI, SHDI and SPLIT is concentrated in major roads, settlement edges, small areas of industrial land and fragmented cultivated land, and low-high clustering is concentrated in fragmented woodland, grassland and orchards. In addition, Due to the fact that village development and construction are more easily restricted by terrain and landforms, especially in JHHR, WAMR and SAMR, villages are mainly built along mountains and arranged near water, and the shape of land use patches is relatively free and flexible. Therefore, there is no obvious land use type and characteristics of spatial coupling relationship between SDI and AWMSI.

**Table 8:** Spatial coupling relationship of village spatial development intensity and landscape pattern.

SDI and LPI, AI, and COHESION	SDI and PD, SHEI, SHDI and SPLIT	Main land use types
High-high clustering		Housing, roads, industrial land, Contiguous cultivated land
	High-high clustering	Main roads, residential land edges, small pieces of industrial land, fragmented cultivated land
	Low-high clustering	Fragmented woodlands, grass land, orchard
Low-high clustering		Contiguous woodland, mountain, river, pond water surface

#### Non-spatial coupling characteristics of village spatial intensity and landscape pattern

The coupling degree and coupling coordination degree of village spatial development intensity and landscape comprehensive index are calculated, so as to explore the non-spatial coupling characteristics between them, and the results are shown in Table 9. The overall coupling degree ranges from 0.6 to 1.0, and the average value of all villages is 0.9517, which means that the whole is in a highly coupled state, indicating a strong interaction and influence between village spatial development intensity and landscape pattern. Among the five regions, the average coupling degree of villages in YRPR is the highest at 0.9941, followed by NAPR, JHHR and SAMR. The average coupling degree of villages in WAMR is the minimum value of 0.8985, showing a trend of “high in plains, medium in hills and low in mountains “. The average coupling degree of the five regions is all greater than 0.8, indicating that they are all in a highly coupled state. The overall coupling coordination degree ranges from 0.3 to 0.9, with an average value of 0.6142, and the whole is in a primary coordination state. The standard deviation of coupling coordination degree is 0.1305, approximately twice that of the coupling degree at 0.0669. The minimum value of coupling coordination degree is 0.3333 and the maximum value is 0.8296, indicating that significant regional differences in coupling coordination. The average coupling coordination degree of villages in NAPR is the highest of 0.7787, followed by 0.7109 of YRPR. The average values of villages in JHHR, WAMR and SAMR are relatively close, all range from 0.50 to 0.55, which are significantly lower than those in plain areas, showing a trend of “high in plains and low in hills and mountains”. In terms of coupling coordination level, the villages in NAPR and YRPR are overall at the intermediate level, while the villages in JHHR, WAMR and SAMR are overall in barely coordinated states.

**Table 9:** Coupling degree and coupling coordination degree of village spatial development intensity and landscape pattern.

Region (county)		NAPR (Mengcheng county)	JHHR (Laian county)	YRPR (Shucheng county)	WAMR (Jinzhai county)	SAMR (Xiuning county)	Total
Coupling degree	Average value	0.9917	0.9484	0.9941	0.8985	0.9259	0.9517
	Standard deviation	0.0078	0.0338	0.0080	0.0859	0.0860	0.0669
	Maximum value	1.0000	1.0000	1.0000	0.9998	0.9994	1.0000
	Minimum value	0.9694	0.8811	0.9692	0.6787	0.6829	0.6787
Coupling coordination degree	Average value	0.7787	0.5447	0.7109	0.5055	0.5312	0.6142
	Standard deviation	0.0318	0.0561	0.0626	0.1095	0.0746	0.1305
	Maximum value	0.8292	0.6564	0.8296	0.7288	0.6946	0.8296
	Minimum value	0.7098	0.4625	0.5838	0.3333	0.3983	0.3333

Furthermore, the coupling coordination types of spatial development intensity and landscape pattern in 100 villages are counted, and the results are shown in Table 10. Coupling coordination types include six types: light disordination, critical disordination, barely coordination, primary coordination, intermediate coordination and good coordination. Among them, the number of villages in light disordination is the least of 4, the number of villages in good coordination is 7, and there are 89 villages in other levels, showing a typical normal distribution. There are 22 villages in a state of disordination (light and critical), accounting for 22%; there are 78 villages in a coordinated state (barely, primary, intermediate, good), accounting for 78%, of which the maximum number of barely coordinated villages is 30 and accounting for 30%. There are only two coupling coordination types of villages in NAPR: intermediate and good, and the coupling coordinated development is the best. The villages in YRPR are all in coordinated development state, but there is only one good coordinated village, which is obviously fewer than that in NAPR. Five villages in JHHR are in critical disordination, and the remaining 15 are in the state of coordinated development. The coupling coordinated development status of WAMR and SAMR are similar, but the number of villages with disordination in WAMR is the largest, as high as 12, which is significantly higher than that of 5 in SAMR, and the coupling coordinated development is the worst. In general, the coupling coordinated development of villages in plain area is the best, followed by villages in JHHR, while villages in mountain area are relatively poor.

**Table 10:** Coupling coordination types of village spatial development intensity and landscape pattern.

Coupling coordination types	Number of villages					
	NAPR (Mengcheng county)	JHHR (Laian county)	YRPR (Shucheng county)	WAMR (Jinzhai county)	SAMR (Xiuning county)	Total
Light disordination	—	—	—	3	1	4
Critical disordination	—	5	—	9	4	18
Barely coordination	—	12	2	4	12	30
Primary coordination	—	3	6	3	3	15
Intermediate coordination	14	—	11	1	—	26
Good coordination	6	—	1	—	—	7

#### Analysis of impact mechanism

The spatial development intensity reflects the interference and impact of human activities on the village landscape system, while landscape pattern is the result of spatial development. Different spatial development intensity leads to different landscape pattern characteristics, so it is necessary to explore the causal relationship between spatial development intensity and landscape pattern. Firstly, the correlation coefficients between the comprehensive landscape index (CLI) and spatial compactness (SC), road density (RD), landscape development intensity (LDI) and spatial development intensity (SDI) are calculated. The results are shown in Table 11. According to the isometric spacing of 0.2, when the absolute value of correlation coefficient is greater than 0.4, it is a strong correlation, and conversely, it is weak. The correlation coefficients between comprehensive landscape index and spatial development intensity, road density and landscape development intensity are all greater than 0.4, which have strong correlations, while the correlation coefficient between comprehensive landscape index and spatial compactness is only 0.0192, indicating very weak correlation. Among the five regions, villages in WAMR show a very strong correlation. Except for spatial compactness, the correlation coefficients of the remaining three indicators are all greater than 0.8. The correlation coefficients of two indicators in JHHR are greater than 0.4, the absolute value of correlation coefficient of only one indicator in NAPR and YRPR is greater than 0.4, and the absolute value of correlation coefficient of all indicators in SAMR are all less than 0.2, and the correlation is very weak. Comprehensive analysis shows that the correlation between village landscape patterns and various spatial development intensity indicators is not high, indicating that there is no obvious causal relationship between them.

**Table 11:** Correlation analysis of village landscape pattern and spatial development intensity.

Region (county)	Correlation coefficients			
	CLI–SC	CLI–RD	CLI–LDI	CLI–SDI
NAPR (Mengcheng county)	−0.6237	0.3475	0.2241	0.1449
JHHR (Laian county)	0.2135	0.3277	0.4677	0.5249
YRPR (Shucheng county)	−0.1403	0.4464	0.3902	0.3522
WAMR (Jinzhai county)	−0.0281	0.8030	0.8143	0.8257
SAMR (Xiuning county)	0.0155	−0.1709	−0.1966	−0.1764
Total	0.0192	0.4496	0.4675	0.4721

In order to explore the impact mechanism of village spatial development on landscape pattern, this study employs multiple linear regression analysis for modeling. Taking comprehensive landscape index as the dependent variable (*y*), spatial compactness (*x*_1_), road density (*x*_2_) and landscape development intensity (*x*_3_) as independent variables, and then the multiple linear regression equations are established. In constructing the multiple regression equation, the forced entry method was adopted, incorporating all independent variables into the equation. The overall evaluation was based on the coefficient of determination (R^2^), the F-statistic for overall significance, and its corresponding p -value. A larger R^2^ value closer to 1 indicates a better fit of the equation, demonstrating that the independent variables explain the dependent variable more comprehensively. Additionally, the significance level p of the F-statistic in the analysis of variance must reach a significance threshold of less than 0.05 for the equation to be considered meaningful. Since the primary goal of this study is to explore the relationship between village landscape patterns and spatial development rather than to make predictions, diagnostic checks for the model primarily focused on multicollinearity (using the Variance Inflation Factor, VIF) and residual independence (using the Durbin-Watson test). The specific results of model construction and diagnostic tests are presented in Table 12.

**Table 12:** Fitting equations of village landscape pattern and spatial development intensity.

Region (county)	Fitting equations	*R* ^2^	F(*p*)	VIF	D-W
NAPR (Mengcheng county)	$y= - 0.4430 - 16.6173{x_1} - 0.0209{x_2}\,+\,0.3579{x_3}$	0.5404	6.2712 (0.0051)	*x*_1_:1.7713 *x*_2_:2.3156 *x*_3_:1.8556	2.1121
JHHR (Laian county)	$y\,=\,0.0133\,+\,3.4302{x_1}\,+\,0.0173{x_2}\,+\,0.0090{x_3}$	0.3566	2.9563 (0.0640)	*x*_1_:1.9502 *x*_2_:2.7575 *x*_3_:1.8763	2.4720
YRPR (Shucheng county)	$y\,=\,0.3625--2.6940{x_1}\,+\,0.0156{x_2}\,+\,0.0410{x_3}$	0.2781	2.0550 (0.1467)	*x*_1_:1.3856 *x*_2_:1.9000 *x*_3_:2.2580	1.9587
WAMR (Jinzhai county)	$y\,=\,0.2675 - 4.6621{x_1}\,+\,0.0209{x_2}\,+\,0.0707{x_3}$	0.7429	15.4118 (0.0001)	*x*_1_:1.2385 *x*_2_:3.4795 *x*_3_:3.6837	1.8088
SAMR (Xiuning county)	$y\,=\,0.4053\,+\,0.4519{x_1}\,+\,0.0035{x_2} - 0.0235{x_3}$	0.0416	0.2313 (0.8732)	*x*_1_:1.2383 *x*_2_:5.2370 *x*_3_:5.3587	1.0117
Total	$y\,=\,0.1933\,+\,0.7242{x_1}\,+\,0.0205{x_2}\,+\,0.0368{x_3}$	0.2307	9.5981 (0.0000)	*x*_1_:1.4013 *x*_2_:4.7847 *x*_3_:4.6143	0.6059

According to Table 12, the p-values of the fitted equations for villages in JHHR, YRPR and SAMR all exceed 0.05, failing the significance test and indicating that the linear relationship of the equations is not established. In contrast, the p-values for the fitted equations of villages in WAMR and NAPR are both less than 0.05, passing the significance test and confirming the linear relationship of the equations. Additionally, the R^2^ values for these equations are both greater than 0.5, specifically 0.7429 and 0.5404, indicating significant overall linear characteristics and satisfactory model fit. In terms of model diagnostic indicators, the VIF values for the fitted equations in WAMR and NAPR are significantly below 10, demonstrating that there is no obvious multicollinearity among the variables, making them suitable for multiple linear regression modeling. Furthermore, the Durbin-Watson test values for the fitted equations are 1.8088 and 2.1121, respectively, both within the range of 0 to 4 and close to 2, suggesting that residuals are largely independent and that the fitted equations meet the basic requirement of residual independence. Overall, the fitted equations for WAMR and NAPR effectively explain the characteristics and patterns of variation in the dependent variables, suggesting a likely causal relationship between landscape patterns and various spatial development intensity indicators. Finally, the fitted equation for all 100 villages in Anhui Province passes the overall significance test with a p-value less than 0.05, and the VIF values indicate no significant multicollinearity. However, the R^2^ and Durbin-Watson test value are relatively low, at only 0.2307 and 0.6059, respectively, indicating poor explanatory power and residual independence. This suggests that the overall linear relationship of the equation is not prominent, and the model fit is relatively modest.

## Discussion

### Mechanism analysis

#### Analysis of the formation of spatial development intensity differences

Under the influence of natural geographical factors, Anhui Province has formed unique terrain and landforms, comprising five geographical regions: NAPR, JHHR, YRPR, WAMR and SAMR, thus leading to the spatial differentiation between village spatial development intensity and landscape pattern. The villages in NAPR have flat terrain, concentrated and contiguous cultivated land, high road density, and the best conditions for development and construction, which are conducive to large-scale agricultural production. At the same time, the villages are densely populated and human activities are more intense, which requires more roads and settlements to carry. Therefore, the village spatial development intensity is generally higher. Villages in YRPR are similar to NAPR in terms of terrain and landforms, and also have good spatial development conditions, but the population density is less than that of NAPR, so the village spatial development intensity is generally smaller than that of NAPR. Villages in JHHR are limited by a certain degree of terrain and landforms, which leads to high development and construction costs, lower land use levels, and lower village spatial development intensity compared to plain areas. Villages in WAMR and SAMR are limited by greater terrain and landforms, which leads to a smaller population size and greater difficulty and cost in land development and utilisation. Taking the road density as an example, the village roads in NAPR are regular, the lines are straight, and the road density is high. The shape of village roads in JHHR and YRPR is often like fishbone or fishnet, and the road density is relatively high. However, the village road density in WAMR and SAMR is obviously low, and the line shape is more complex, which is clearly not conducive to development and construction. As a result, the village spatial development intensity in both regions is generally low.

#### Analysis of the formation of landscape pattern differences

In terms of landscape pattern, the village population density and human activities in NAPR and YRPR are high, which leads to a greater degree of disturbance to the village natural landscape. The scale of construction land is larger, and the types of land use are more diverse, further resulting in a significant increase in landscape fragmentation. The villages in WAMR and SAMR are located in mountainous areas with high altitudes and abundant forest resources. The terrain and landforms impose significant restrictions on development and construction, resulting in fewer human activities and less disturbance to the village natural landscape. Therefore, the construction land scale is small, and the spatial development intensity is low. As a result, villages in WAMR and SAMR can retain large-scale original ecological rural landscape elements and the lowest degree of landscape fragmentation. Villages in JHHR are located between plains and mountainous villages in terms of development and construction difficulty, human activities and spatial development intensity. The village natural landscape is disturbed at a medium level, and the degree of landscape fragmentation is relatively low. In terms of spatial coupling relationship between village spatial development intensity and landscape pattern, the greater the road density, the more settlements and the closer the distance to roads and settlements, the greater the spatial development intensity and the greater the landscape fragmentation, which is more conducive to form a spatial coupling relationship of high-high clustering. On the contrary, it is conducive to form a spatial coupling relationship of low-high clustering.

### Development suggestions

#### Spatial development suggestions

Based on the spatial differentiation and coupling relationship between village development intensity and landscape patterns in Anhui Province, it is recommended that the province prioritize the coordinated and unified approach to land development and conservation at the overall level. For spatial development, activities should align with land-use planning guidelines, confining development and construction within village boundaries and following principles of efficient and intensive land use. This includes maximizing the potential of existing construction land to improve land use efficiency in villages. In terms of spatial conservation, it is essential to strictly protect permanent basic farmland and uphold ecological protection boundaries to fundamentally safeguard the natural ecological mechanisms of villages. Additionally, all levels of natural and cultural elements should be preserved according to the protection lines designated in the national spatial plan. Specifically, for the dominant coupling types identified as “high-high” and “low-high” spatial couplings, the following suggestions are proposed.

In high-high clustering areas, where spatial development intensity is high, landscape heterogeneity and fragmentation levels are also elevated, posing challenges for meeting the requirements of efficient and compact land use in villages, which hinders sustainable development. Moving forward, it is essential to strengthen land use planning and area consolidation in these regions to reduce uncoordinated planning and construction. Boundaries for main roads and residential areas should be clearly defined to minimize fragmented development patterns. For industrial and residential land, a concentrated layout should be prioritized, based on a rational determination of land scale and location. Fragmented farmland could be consolidated, where feasible, to create more organized and concentrated agricultural areas.

In low-high clustering areas, spatial development intensity is relatively low, yet landscape characteristics also exhibit high fragmentation and heterogeneity, reflecting insufficient conditions for village development and construction. These areas are crucial for ecological conservation and ecosystem restoration. Future efforts should focus on protecting ecological lands, such as forests, grasslands, and orchards, within these areas. Spatial development activities should be strictly controlled, emphasizing ecological restoration and conservation, enhancing environmental protection facilities, and ensuring no degradation in environmental quality.

#### Non-spatial development suggestions

The villages in NAPR and YRPR have high spatial development intensity, good development foundation, and concentrated and continuous high-quality cultivated land, which are suitable for the development of modern agriculture. In the future, the villages in NAPR and YRPR should make full use of the existing advantages and conditions of development and construction to actively develop modern agriculture and promote the comprehensive revitalisation of rural areas The villages in JHHR not only have good conditions for development and construction, but also have good natural ecological resources, which means that modern agricultural development can be organically combined with the creation of natural ecological countryside, thus creating a rural landscape with regional characteristics. The village natural ecological conditions in WAMR and SAMR are superior, and the village original ecological landscape is well preserved. It should make full use of the rich mountain and woodland ecological resources to actively develop green agriculture and ecotourism. At the same time, it is necessary to conserve and intensively utilise valuable land resources, control village development and construction intensity, avoid the disorderly expansion of construction land, protect village natural ecological landscape, and ultimately achieve the coordination and unity between development and construction and ecological protection.

### Analysis of the representativeness of research findings

#### Representativeness of the study area

In addition, the regional representativeness of the research findings warrants further discussion. Given the large number of villages in Anhui Province, conducting a comprehensive study of all villages would be highly challenging. The most practical approach is to select representative villages for investigation, which serves as the basis for this study. During the selection of village samples, the study first chose an equal number of villages from different geographical divisions. Subsequently, within each division, villages were selected to represent diverse spatial forms, varying sizes, and distinct locational characteristics. This approach aimed to encompass as many different types of villages as possible, ensuring that the selected 100 villages could maximally reflect the overall characteristics of villages in Anhui Province. As a result, the research findings are well-suited to represent the general conditions of villages in the province. From a broader spatial perspective, China features highly diverse topography and geomorphology, including plains, mountains, and hills. Anhui Province, with its highly similar terrain and landforms, serves as a typical representation of China’s topographical features. In this context, this study provides valuable insights for exploring the relationship between spatial development intensity and landscape patterns in Chinese villages. Furthermore, villages in other countries are also predominantly distributed across plains, mountains, and hilly regions. The village samples in this study, drawn from plains, mountains, and hills, can offer comparative samples and serve as a valuable reference for studying spatial development and landscape patterns of villages in other nations.

#### Representativeness of the study samples

Finally, it is worth noting that potential biases may exist in the selection of samples for this study. Certain special types of villages, such as those with historical and cultural significance, those located within ecological conservation zones, or villages in urban suburbs, may not have been included in the study samples. Additionally, Anhui Province’s unique natural geographic conditions, resource environments, and socioeconomic development characteristics exert distinct influences on village spatial development and landscape patterns. The research findings are the reflection of these unique influences.

## Conclusions and research prospects

### Conclusions

Existing studies mainly focus on individual aspects of spatial development or landscape patterns, with limited comprehensive research on their coupling relationship. Additionally, previous studies have primarily focused on urban and regional macro-scales, with limited theoretical and empirical research at the micro-scale level of villages. This study provides a method and case study to address these research gaps. It constructs an integrated framework to evaluate the spatial differentiation and coupling relationship between village spatial development intensity and landscape patterns, with specific application to 100 villages in Anhui Province, China. The findings contribute to the theoretical and methodological research system for spatial development and landscape patterns. They also offer valuable practical applications for village land development and conservation, landscape pattern optimization, and village planning.

Empirical research shows that the spatial development intensity and landscape pattern of 100 villages in Anhui Province have obvious regional differences. The village spatial development intensity in NAPR is the highest, followed by YRPR and JHHR, and the village spatial development intensity in WAMR and SAMR is relatively low. The landscape pattern of villages in NAPR show high aggregation and high fragmentation, villages in JHHR show low fragmentation and high connectivity, villages in YRPR show high fragmentation and low aggregation, and villages in WAMR and SAMR show low fragmentation and low aggregation. The natural ecological landscape of villages in plain area is greatly disturbed by human activities, and the degree of landscape fragmentation is high. On the contrary, the natural ecological landscape of villages in hilly and mountainous area is less disturbed by human activities, and the natural landscape pattern is more preserved. The spatial coupling relationship between village spatial development intensity and landscape pattern is mainly characterised by high-high clustering and low-high clustering. Different clustering corresponds to different land use types. At the same time, the spatial coupling relationship of the villages in the five regions also has certain differences. As for non-spatial coupling relationship, the coupling coordinated development of villages in the plain area is the best, followed by villages in hilly area, and villages in mountainous area is relatively the worst. The analysis of the impact mechanism shows that there is no significant causal relationship between village landscape pattern and each spatial development intensity index. There is only a significant multi-linear relationship between village landscape pattern and multiple spatial development intensity indicators of villages in WAMR and NAPR. Affected by the dual influence of natural conditions and human activities, village spatial development intensity, landscape pattern and their coupling relationships have all formed significant regional differences. Finally, the study proposes targeted countermeasures and suggestions.

### Research prospects

Future research should focus on the following aspects. For instance, the analysis of village spatial development intensity in this study primarily considers material spatial development, with limited attention to economic and social factors. Incorporating these dimensions would provide a more comprehensive understanding. Additionally, exploring the temporal evolution characteristics of spatial development intensity and landscape patterns represents another important direction for future research. For another example, there are still some limitations in the calculation of the coupling degree and coupling coordination degree models. In Eq. (9), determining the weights of different systems poses a challenge. This study assumes equal weights for the systems, but there may be more appropriate weight values. Clearly, different weights will yield different results. Therefore, adopting scientifically robust methods to calculate weights will be a key focus for future model applications and similar studies. In addition, nonlinear modeling analysis can also be carried out on the impact mechanism of village spatial development intensity and landscape pattern, so as to reveal the impact mechanism more deeply. Finally, the selection of sample villages needs further refinement. Increasing the number of village samples is crucial to better capture the overall characteristics and patterns of villages in the study area. Moreover, including a greater variety of village types would help to more comprehensively reveal the spatial development and landscape pattern characteristics of different types of villages. For example, adding village samples with historical and cultural significance could offer valuable insights, as the need to preserve cultural heritage in these villages may lead to distinct spatial development and landscape pattern dynamics.

The study of village spatial development intensity and landscape pattern is an important part of the current rural research system, and it is still in the process of continuous deepening and improvement. This study hopes to provide a certain reference for village spatial development and protection in Anhui Province and other areas. In the future, more sample villages can be selected for in-depth exploration, which can play a more scientific guiding role in achieving comprehensive rural revitalization.

## Data Availability

Data is provided within the manuscript. Those wishing to request data from this study can contact Lihua Chen.
